# Microwave Imaging of Human Forearms: Pilot Study and Image Enhancement

**DOI:** 10.1155/2013/673027

**Published:** 2013-08-19

**Authors:** Colin Gilmore, Amer Zakaria, Stephen Pistorius, Joe LoVetri

**Affiliations:** ^1^TRTech Inc., Winnipeg MB, Canada R3T 6A8; ^2^Department of Electrical and Computer Engineering, University of Manitoba, Winnipeg MB, Canada R3T 5V6; ^3^Department of Physics and Astronomy, University of Manitoba, Winnipeg MB, Canada R3T 2N2; ^4^Medical Physics, CancerCare Manitoba, Winnipeg MB, Canada R3E 0V9

## Abstract

We present a pilot study using a microwave tomography system in which we image the forearms of 5 adult male and female volunteers between the ages of 30 and 48. Microwave scattering data were collected at 0.8 to 1.2 GHz with 24 transmitting and receiving antennas located in a matching fluid of deionized water and table salt. Inversion of the microwave data was performed with a balanced version of the multiplicative-regularized contrast source inversion algorithm formulated using the finite-element method (FEM-CSI). T1-weighted MRI images of each volunteer's forearm were also collected in the same plane as the microwave scattering experiment. Initial “blind” imaging results from the utilized inversion algorithm show that the image quality is dependent on the thickness of the arm's peripheral adipose tissue layer; thicker layers of adipose tissue lead to poorer overall image quality. Due to the exible nature of the FEM-CSI algorithm used, prior information can be readily incorporated into the microwave imaging inversion process. We show that by introducing prior information into the FEM-CSI algorithm the internal anatomical features of all the arms are resolved, significantly improving the images. The prior information was estimated manually from the blind inversions using an *ad hoc* procedure.

## 1. Introduction

Microwave imaging (MWI) is an alternative imaging modality that promises several advantages over more established modalities such as X-ray, ultrasound, or MRI. Advantages include low cost, use of safer nonionizing radiation, the ability to image bulk-electrical tissue properties, and the ability to provide functional imaging without the use of contrast agents [1]. Microwave imaging applications have primarily focused on breast cancer [2–7], although extremity (arm and leg) imaging has also received attention [1, 8–12]. While standard X-ray imaging gives reliable indication of bone injury, some researchers have indicated that diagnosing the condition of soft tissue is important for the final outcome of treatment [8], and microwaves may potentially be used to assess the soft tissue component of an injured extremity. Despite the potential advantages of microwaves as an imaging modality, the technology has not yet seen widespread use in clinics outside of research labs (e.g., the largest study involves 400 volunteers [13]).

We believe that the best argument for the use of microwave imaging is that it promises to fill a niche within the medical imaging world, providing a nonionizing, inexpensive imaging modality which is capable of imaging soft tissue contrast. The three most common medical imaging modalities are ultrasound, X-rays (both planar and CT), and magnetic resonance imaging (MRI). Ultrasound is inexpensive and nonionizing but has trouble distinguishing between soft tissues. Planar X-rays are inexpensive and give only a small dose of ionizing radiation but also struggle with imaging soft tissue contrast. X-ray CT is somewhat inexpensive and has good soft tissue contrast imaging capabilities but gives a significant dose of ionizing radiation per scan. (This radiation is particularly important for children: a single abdominal helical CT in young female children results in 1 in 1000 risks of fatal cancer later in life [14].) MRI is nonionizing, offers excellent soft tissue contrast imaging, but scanners are expensive to buy and maintain, and a single scan can take over an hour.

Due to the significant differences in permittivity between soft/hard tissues and other bodily fluids [15], its use of nonionizing radiation, the ability to provide quantitative images, and relatively inexpensive hardware, microwave tomography could become a viable modality in medical imaging. In addition to the aforementioned breast cancer and soft tissue injury imaging, there are also opportunities in cyst fluid identification, screening/monitoring programs for degenerative muscular disorders (e.g., muscular dystrophy), lung carcinomas, stroke identification, and others that to date have not received much attention [16].

Despite this promise, initiating further clinical interest requires experimental images of live tissue, showing that the technology is capable of providing clinically relevant images. However, imaging live humans is more challenging than imaging phantoms as volunteer safety, movement, variations in size, and the presence of complex tissues, not entirely predicted by simplified phantoms, need to be considered. (It has often been noted that the gap between theory and practice is larger in practice than it is in theory.) We argue that the largest barrier to microwave tomography (MWT) is actually the lack of clinical images available to be taken to the clinical professionals who regularly read anatomical images (e.g., radiologists). Once these images have been generated, these professionals can further direct the technology.

In this work, we take a large step towards a general 2D clinical imaging system by presenting (to the best of our knowledge) the first study of microwave limb imaging with multiple individuals (either human or animal). We have elected to image the forearms of 5 human volunteers with varying ages and (importantly) varying levels of adipose tissue. The quantitative microwave images are supplemented with collocated (but not simultaneous) MRI images of the same limb. This study outlines the capabilities and some of the remaining challenges for microwave imaging of human tissues, and provides useful information on *in vivo* permittivity measurement. In addition, an enhancement for the microwave imaging reconstruction quality is introduced by incorporating prior information about the forearm's adipose layer into the inversion algorithm utilized in this work.

The paper is arranged as follows. An overview of the experimental system utilized in our study is outlined in Section 2. The considered problem's mathematical formulation as well as a description of the inversion algorithm is given in Section 3. Methods related to performing various measurements are described in Section 4. The preliminary microwave imaging results of the volunteers along with the MRI scans are presented in Section 5. Enhancements to the MWI results by the use of prior information are shown in Section 6. Finally, the paper is closed by a brief conclusion in Section 7.

## 2. System Overview

**Figure 1:**
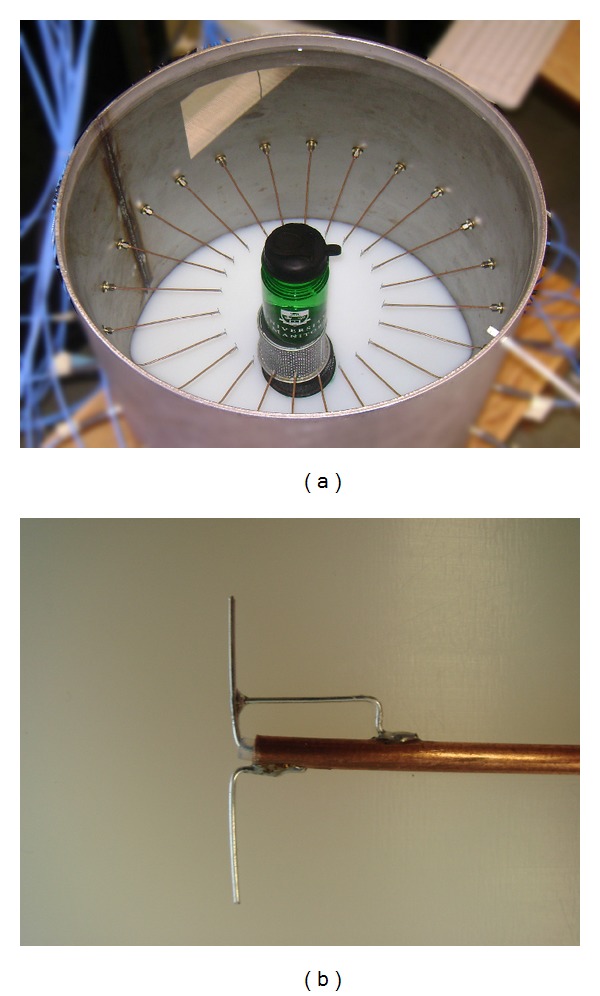
(a) The MWI system metallic enclosure with a glycerol-/water-based imaging phantom. (b) A dipole antenna with quarter wavelength balun.

The microwave imaging system consists of a network analyzer (Agilent PNA network analyzer E8363) connected to a 2 × 24 matrix switch (Agilent 87050A-K24). Twenty-four dipole antennas with a quarter wavelength balun are arranged at even intervals of 15° in a circular array at the midpoint height along the inside of a metallic cylinder. This system is similar to a previously described system [17] and has also been presented in [18]. The antennas are located at a radius of 9.4 cm from the center of the chamber. The enclosure has a radius of 22.4 cm and is filled, to a height of 44.4 cm, with the matching fluid.  Figure 1(a) shows a photograph of the MWI system metallic enclosure with a tissue-mimicking imaging phantom in the imaging region, while a picture of a single dipole antenna is shown in Figure 1(b). The total volume of fluid in the chamber is approximately 70.0 L. The system is capable of imaging from approximately 800 MHz to 1.2 GHz in a salt/deionized water background.

The experimental apparatus is controlled via a computer workstation which is connected through a local-ethernet device. In-house developed software is used to collect the dataset for each desired image. Data for 20 discrete frequencies are acquired in slightly less than 1 minute. The number of measurements per frequency is 24 × 23.

### 2.1. Matching Fluid

**Figure 2:**
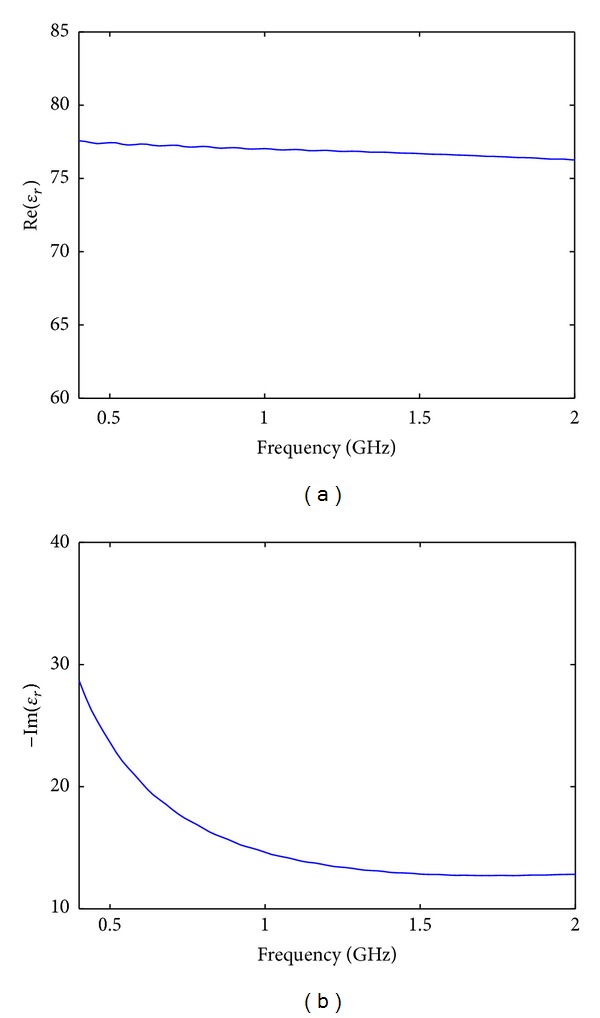
(a) Real and (b) imaginary components of the complex relative permittivity used in our MWI system as matching fluid.

In general, biomedical microwave imaging requires the use of a matching fluid [19]. Our system uses a fluid of deionized water and table salt. The salt is added in order to introduce loss into the matching fluid, which reduces the modeling error: the mismatch between the assumed computational model and the physical experiment. However, adding too much salt can decrease the signal to noise of the measurement to unacceptable levels. We have previously determined that an appropriate amount of salt is 2.5–4.5 grams per liter [18], and for this study we use approximately 3.1 grams/liter. A plot of matching fluid complex relative permittivity is shown in Figure 2. At a frequency of 1 GHz, the relative permittivity of the matching fluid is *ϵ*_*r*_ ≈ 77 − *j*15.

## 3. Mathematical Formulation

We consider a two-dimensional (2D) mathematical formulation with the electric field polarized along the longitudinal *z*-axis of the problem. A time-harmonic field of frequency *f* is assumed. An object of interest (OI) within an imaging domain *𝒟* is immersed in an inhomogeneous background medium contained within an imaging chamber. The space within the imaging chamber *Ω* is confined by a boundary Γ. The OI and the background medium are assumed to be nonmagnetic with permeability *μ* = *μ*_0_, the free space permeability. At a 2D position vector $r=x\hat{x}+y\hat{y}$, the OI's relative complex permittivity is given as

(1) ϵr(r)=ϵr′(r)−jϵr′′(r)=ϵr′(r)−jσeff  (r)(2πfϵ0),

where *j* ^2^ = −1, *ϵ*_*r*_′ and *σ*_eff  _ are, respectively, the real relative permittivity and the effective conductivity of the OI, and *ϵ*_0_ is the permittivity of free space. Note that *ϵ*_*r*_′ and *σ*_eff  _ are frequency dependent. Given the complex relative permittivity of the background as *ϵ*_*b*_(**r**), the contrast of the OI within *𝒟* is defined as

(2) χ(r)≜(ϵr(r)−ϵb(r))ϵb(r),

whereas outside *𝒟χ* = 0.

The chamber is illuminated successively by one of the *T* transmitters, producing an incident field *E*_*t*_^inc^(**r**) defined as the field produced by transmitter *t* in the presence of the inhomogeneous background medium and in the absence of the OI. The transmitters are assumed to be 2D electric point sources (lines sources in 3D) with the incident field produced by transmitter *t* calculated as

(3) Etinc(r)=1j4H0(2)(kb|r−rt|).

Here *H*_0_^(2)^ is the zeroth-order Hankel function of the second kind, $k_{b}(r)=2\pi f\sqrt{\mu_{0}\epsilon_{0}\epsilon_{b}(r)}$ is the background medium wavenumber, and **r**_*t*_ is the location of transmitter *t*.

In the presence of the OI, the resultant field in the chamber is the total field *E*_*t*_^tot^(**r**). Both the incident and total fields are measured at *R* receiver locations positioned on a measurement surface *𝒮*. The scattered field, due to the difference in electrical properties between the OI and the background medium, is defined as *E*_*t*_^sct^(**r**)≜*E*_*t*_^tot^(**r**) − *E*_*t*_^inc^(**r**) and is governed by the scalar Helmholtz equation

(4) ∇2Etsct(r)+kb2(r)Etsct(r)=−kb2(r)wt(r),

where *w*_*t*_(**r**)≜*χ*(**r**)*E*_*t*_^tot^(**r**) is the contrast source.

The boundary-value problem, defined by (4) as well as boundary conditions, is solved using FEM [20]. The discretization of the MWI problem using FEM is detailed in [21].

### 3.1. Inversion Algorithm

MWI is associated with an inverse scattering problem, which can be solved using optimization-based algorithms that attempt to minimize a cost functional. The minimization optimizes variables that relate to the properties of the OI. Depending on the algorithm's outcome, MWI techniques may be generally split into two categories: quantitative (tomographic) techniques and qualitative (radar-based) techniques. Tomographic techniques (e.g., [17, 22–25]) use a limited number of discrete frequencies and provide simultaneous quantitative images of the dielectric constant and effective conductivity at those frequencies. On the other hand, radar techniques (e.g., [26–29]) use a large number of discrete frequencies (or time-domain pulses) and provide a single qualitative image of the “reflectivity” of the OI. We use the quantitative tomographic reconstruction technique in this work.

In this paper, the experimental data are inverted using the contrast source inversion algorithm formulated using the finite-element method (FEM-CSI) [30, 31]. FEM-CSI offers the ability of performing the inversion on an unstructured grid of triangles with varying mesh density; the density of the mesh elements can be varied so as to decrease the algorithm's computational complexity without compromising the reconstruction quality [32]. In addition, FEM-CSI eases the incorporation of prior information as inhomogeneous background; as will be demonstrated in Section 6, this feature is used to improve the quality of the reconstructions. The FEM-CSI algorithm is implemented in MATLAB and takes approximately 30 minutes per image on a machine with two 2.8 GHz quad-core processors.

The outcome of the inversion algorithm is enhanced by utilizing a balanced multiplicative regularizer [33, 34]. The weighted *L*_2_-norm total variation regularizer has edge-preserving capabilities [35], as well as the ability to correct the imbalance that may exist between the real and imaginary components of the OI's relative permittivity.

## 4. Methods

**Figure 3:**
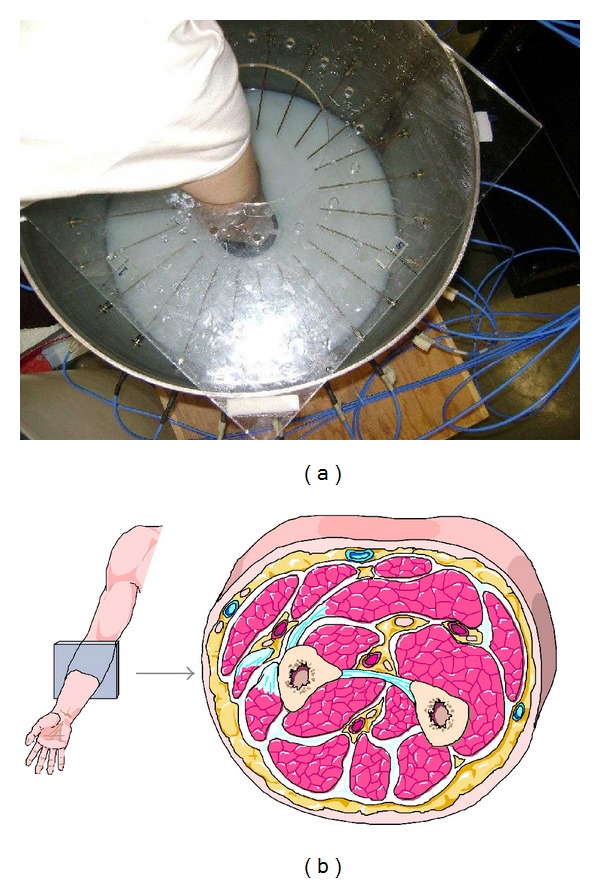
(a) A volunteer's arm inside the microwave imaging system. (b) Forearm anatomy, with the approximate location of microwave imaging plane.

All research was carried out at the University of Manitoba under a University of Manitoba Biomedical Research Ethics Board approved protocol. Volunteer inclusion criteria were that the volunteers were over 18, without implants or tattoos, and not pregnant. Five individuals were used in this pilot study. Each volunteer had one forearm imaged of his/her choice (left/right). The sex, age, and left/right forearm information is listed in Table 1. A Plexiglas lid on the chamber, with a hole in the center, was used to help volunteers keep their arms supported and minimize motion during data collection. Each volunteer was asked to obtain a comfortable position and then hold their arm as still as possible during the data collection. Data collection time was slightly less than 1 minute for all frequencies. A photograph of a volunteer's arm in the imaging system is shown in Figure 3(a). For reference, we have included a diagram of the arm's anatomical structure at the approximate spot of the imaging plane in Figure 3(b).

For each volunteer, 23 × 24 data points (*S*-parameter measurements) were collected in 100 MHz steps from 0.4 to 2 GHz, although not all frequencies are suitable for imaging. For this imaging system and antennas, we have empirically found that the best imaging frequencies are between 0.8 and 1.2 GHz, mostly due to the antennas radiating efficiently in this range. Prior to imaging of each volunteer, data were also taken of the empty fluid-filled chamber and of a 3.5-inch diameter metallic cylinder centered in the chamber. The empty chamber measurements constitute measurements of the incident field, and the metallic cylinder is used for calibration. Details of the calibration method may be found in [17, 18, 36]. 

### 4.1. Noise Metric

To provide an estimate of the signal to noise ratio of the experimental microwave data, we define a noise metric (used previously in [18]) as

(5) N=1ni,j∑(i,j)  pairs||ui,jsct−uj,isct||||ui,jsct||,

where *u*_*i*,*j*_^sct^ is the calibrated scattered field measurement, and the sum is taken over all transmit-receive pairs of *i* and *j*, and *n*_*i*,*j*_ is the total number of transmit-receive pairs. We justify this metric since from reciprocity *u*_*i*,*j*_ = *u*_*j*,*i*_ and anything else in the data must be noise. This metric (most importantly) is capable of detecting volunteer movement, as the measurements *u*_*i*,*j*_ and *u*_*j*,*i*_ can be up to 1 minute apart. If the metric is significantly higher for a volunteer than for a stationary phantom target, the volunteer was likely moving enough to create image artifacts, and we recollect the data. The metric also provides a measure of the thermal measurement noise. It does not account for modeling error (the differences between our assumed computational model and physical measurement system).  Table 2 presents the noise metric, *N*, for each inverted data set.

### 4.2. MRI

To provide a baseline of each volunteers anatomy, each volunteer was also imaged with a 0.2 T Esaote E-scan XQ MRI, using a forearm coil. This occurred less than 1 hour after the collection of the microwave data. A standard T1 gradient echo protocol was used to obtain transverse MR images in the same plane as the microwave data. To provide an approximate landmarking location for the transverse plane, a vitamin E capsule was affixed with medical tape to the arm at approximately the same height as the microwave imaging plane. The capsule was not present during microwave imaging.

We have attempted to manually coregister the MRI and microwave images. The axes for each volunteer's images are identical, but we have rotated the microwave data to obtain a similar arm orientation between the two modalities. However, the accuracy of the coregistration is limited since the volunteers arm and body positions were different in the MRI and microwave imaging systems. In the microwave system, volunteers were standing and the arm held vertically with the hand clenched in a fist resting on a pad at the bottom of the chamber. In the MRI system the volunteers were supine and the arm held horizontally, with the forearm resting on the MRI forearm coil and the hand in an open resting position. Further, support pads were inserted into the MRI coil, which added varying degrees of compression to the forearm soft tissue. The resultant soft tissue deformations between the two modalities prevent ideal coregistration.

## 5. Experimental Results

**Figure 4:**
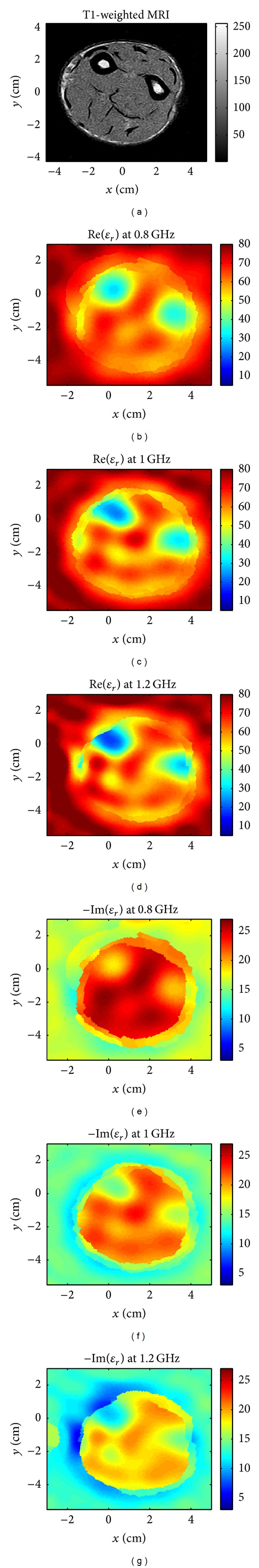
Volunteer 1: (a) the T1-weighted MRI image. MWI reconstructions of the relative complex permittivity real and imaginary components at (b), (e) 0.8 GHz, (c), (f) 1 GHz, and (d), (g) 1.2 GHz.

**Figure 5:**
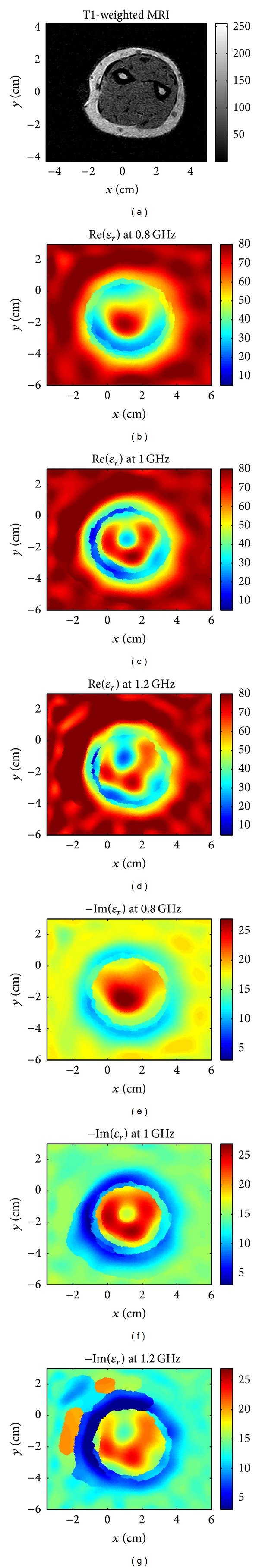
Volunteer 2: (a) the T1-weighted MRI image. MWI reconstructions of the relative complex permittivity real and imaginary components at (b), (e) 0.8 GHz, (c), (f) 1 GHz, and (d), (g) 1.2 GHz.

**Figure 6:**
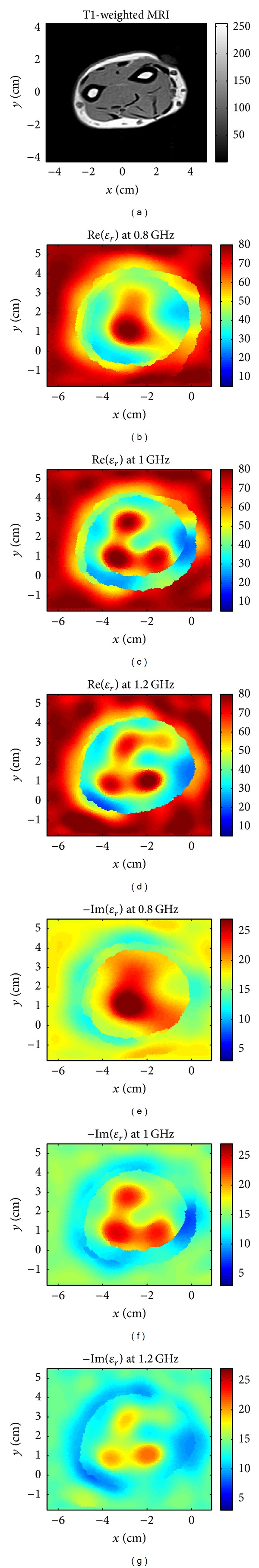
Volunteer 3: (a) the T1-weighted MRI image. MWI reconstructions of the relative complex permittivity real and imaginary components at (b), (e) 0.8 GHz, (c), (f) 1 GHz, and (d), (g) 1.2 GHz.

**Figure 7:**
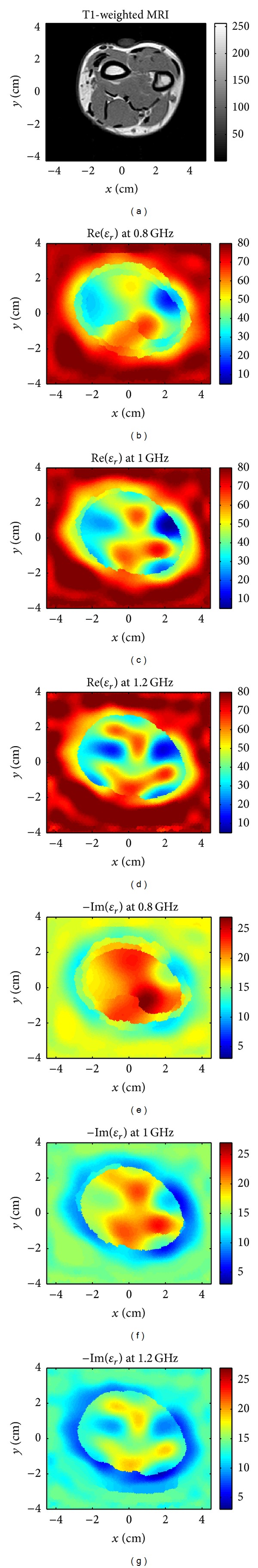
Volunteer 4: (a) the T1-weighted MRI image. MWI reconstructions of the relative complex permittivity real and imaginary components at (b), (e) 0.8 GHz, (c), (f) 1 GHz, and (d), (g) 1.2 GHz.

**Figure 8:**
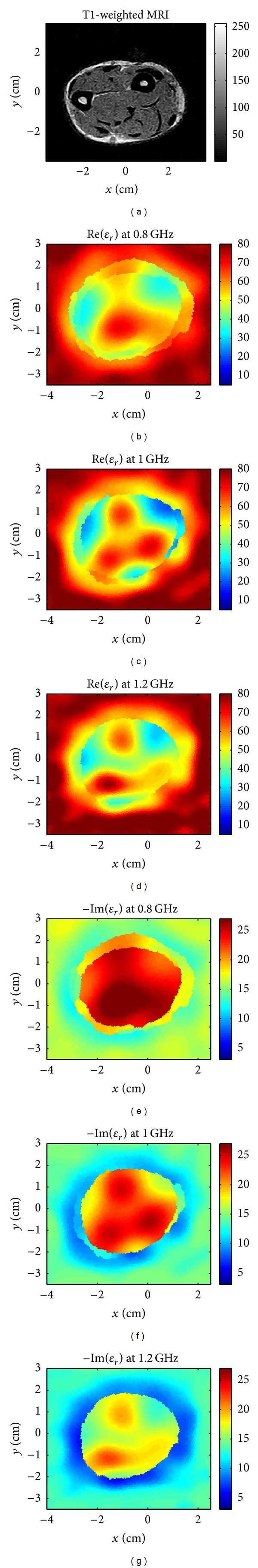
Volunteer 5: (a) the T1-weighted MRI image. MWI reconstructions of the relative complex permittivity real and imaginary components at (b), (e) 0.8 GHz, (c), (f) 1 GHz, and (d), (g) 1.2 GHz.

For each volunteer, we present a figure with the MRI image and a series of microwave-based images of the complex relative permittivities at 0.8, 1, and 1.2 GHz. Figures 4 to 8 show the results for all 5 volunteers. All color scales are kept identical for real and imaginary parts of the relative permittivity throughout this work.

In Table 3, we list the maximum thickness of the adipose layer for each volunteer, as measured from the MRI. Table 3 also presents the width of the arm, from a transect taken along the line defined by the center of the two bones for each volunteer, and the ratio of the width to maximum adipose thickness (the width-to-adipose ratio). Furthermore, using the MWI reconstructions we added also to Table 3 our estimates (to the nearest whole number) of the relative permittivities inside the muscle region of the microwave reconstruction for all the 5 volunteers. These estimates were obtained by identifying the muscle in the MRI and then determining the pixels in the microwave image within the muscle region that had the lowest and highest values. These regions were, in some cases, difficult to select, so the results in Table 3 are estimates only.

### 5.1. Discussion

**Figure 9:**
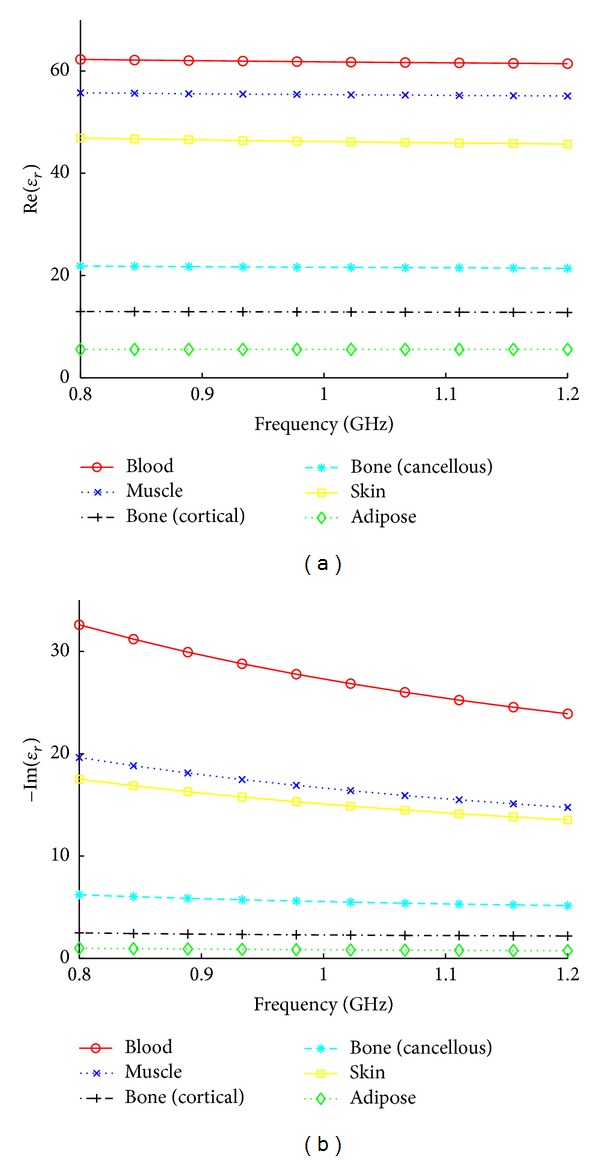
Relative permittivities of various human tissues from 0.8 to 1.2 GHz using the model in [15].

As a companion for this discussion, we have included tissue relative permittivities taken from the literature [15, 37] in Figure 9. The measurements from the literature were taken *ex vivo*. However, many *in vivo* tissue relative permittivities are not the same as those measured *ex vivo*, for example, [38, 39]. These differences are due to temperature changes, tissue dehydration, and devascularization of the excised tissues [38]. As our microwave imaging system measures *in vivo*, the relative permittivities presented in Figure 9 should not be taken as the exact expected values, rather as a general guideline.

As can be seen from Figures 4, 5, 6, 7, and 8, the quality of the microwave reconstructions is improved for volunteers with less adipose tissue with the two arm bones being visible in volunteers 1, 4, and 5. This corresponds to the three smallest maximum adipose thicknesses (3.9, 7.0, and 4.3 mm) and the highest width-to-adipose ratios (16.6, 9.0, and 12.3 mm, as taken from the data). For volunteers 2 and 3, which have the thickest adipose tissue and highest width-to-adipose ratios, the two bones are not readily visible in the microwave images, and in the real part of the permittivity there are significant artifacts inside the arm. For example, in the real part of the 1 GHz reconstructions there are regions with *ϵ*_*r*_ ≈ 80, which is not expected inside the arm.

It is clear that the image quality is strongly and inversely dependent on the thickness of the subcutaneous adipose layer. For volunteer 1 (Figure 4), both bones are visible at all three frequencies. The real part of the permittivity of the muscle tissue varies between 56 and 67 (approx.) for all three frequencies, while the average imaginary permittivity of the muscle drops steadily as the frequency increases (approximately 28, 23, and 21 for 0.8, 1, and 1.2 GHz). This agrees with the trends seen in the permittivity values in the literature: from Figure 9, we expect the real part of the permittivity of muscle and blood to be relatively constant across this frequency range, while the imaginary part of the permittivity is expected to decrease as the frequency increases. As noted, we do not expect exact agreement between the literature and measured values because of the differences between *in vivo* and *ex vivo* measurements.

The noise metric results for the experimental data in Table 2 show that image quality differences between volunteers are not due to differences in signal to noise levels, at least with respect to volunteer movement and thermal noise. This is clear because the best images (volunteer 1) have a noise metric higher than the poorest images (volunteer 2) for all 3 frequencies presented. Of course there could still be differences in modeling error between volunteers (e.g., there could be more 3D artifacts from a given volunteer's arm), but this is not quantifiable. We expect that the differences seen in the noise metric between the volunteers are due to minor volunteer movement during the measurement procedure.

With respect to limb imaging in the literature, we know of only one other human forearm [40], collected with a 64-antenna system at 2.33 GHz, which we have previously inverted in [41]. Our previous imaging results compare well with our images from volunteer 1 in this study, despite lower frequency and significantly fewer antennas in our system. Although we cannot know for certain, we suspect that the volunteer in [40] had low adipose content in his/her forearm.

Another limb imaging in the literature considers swine limbs [1, 9]. In [9], the swine limb is excised, and qualitatively the 2D images in [9] are similar to our images for volunteers 1, 4, and, 5 in that (a) the bone tissue is readily visible; (b) the exterior of the limb is well-defined; and (c) similar muscle permittivities were found. With respect to the live-swine forearm images in [1], our images are qualitatively better in that (a) the exterior shape of the limb is readily visible and (b) we seem to see muscle permittivities which more closely match the expected values (we do note that [1] is primarily concerned with functional, not structural, imaging, and this may have affected system design, matching fluid selection, etc.).

Although images are not shown here, we have tried marching-on-frequency inversion with the experimental data, which resulted in no visible improvement in images (we struggled to see any difference by eye). We speculate that the use of a greater frequency range (e.g., 1 GHz to 6 GHz, [42]) would lead to improvements and note that this result is only applicable to our system.

## 6. Prior Information Incorporation

The use of prior information to enhance the quality of the reconstructions in MWI has been investigated previously in the literature. We categorize the various methodologies into two groups: the first category uses prior information about the *structure* of the OI being imaged [43, 44], whereas the second incorporates information about the *electrical properties* of the OI [45]. Hybridization of the two categories is also possible. For example, a hybrid technique may incorporate the prior information about the OI structure and properties into the mathematical operator that describes the physics of the inverse scattering problem [31, 46]. In this section, we make novel use of FEM-CSI ability of incorporating estimated prior information as an inhomogeneous background in its forward scattering operator.

### 6.1. Estimation and Inversion

While incorporating prior information into FEM-CSI is relatively simple, the challenge becomes developing a methodology of accurately estimating the nonuniform adipose layer. Although non-MWI-based techniques are certainly possible [47, 48], in this paper the estimation of each volunteer's forearm adipose layer from the blind inversion images is done manually. That is, we manually identify three different regions from the blind inversion results: the outer background, a single adipose layer, and an inner muscle region. Due to the *ad hoc* nature of estimating the regions, different people may get different estimates; the estimations used in this paper were obtained by a *trained eye* that has been studying synthetic models of the forearm to understand how the location of the adipose layer can be identified from blind inversion results. Such investigations are best performed using synthetic model and data. Preliminary results using automated procedures have been investigated by our group [49] but are beyond the scope of the paper. In this section, the goal is to show that *substantial* improvements can be obtained even via an *ad hoc* technique of estimating the prior information.

The manual steps which are used to create the inhomogeneous background for each volunteer's forearm can be summarized as follows.

- A blind inversion of the experimental dataset is performed at 1 GHz using the balanced MR-FEM-CSI algorithm.
- A nonuniform adipose layer location is estimated within the resulting image using our experience of which the region within the blind result might be adipose. For example, the estimation of the adipose layer is largely obtained from viewing the imaginary part of the blind reconstruction. While the adipose layer was being estimated, we did not use the associated MRI image.
- The unstructured mesh nodes within the identified layer are assigned the complex relative permittivity of *ϵ*_*r*_ = 10 − *j*1, which is an estimated value taken from published values of permittivity for adipose tissue [15].
- The nodes outside the adipose layer are assigned the permittivity of the salt-water matching medium.
- The nodes enclosed by the adipose layer are assigned a relative permittivity value of *ϵ*_*r*_ = 50 − *j*20, an estimated value for the muscle.
- The inversion algorithm is rerun using the estimated prior information as a inhomogeneous background. The same estimated inhomogeneous background is used to invert the data at the three frequencies: 0.8, 1, and 1.2 GHz.

**Figure 10:**
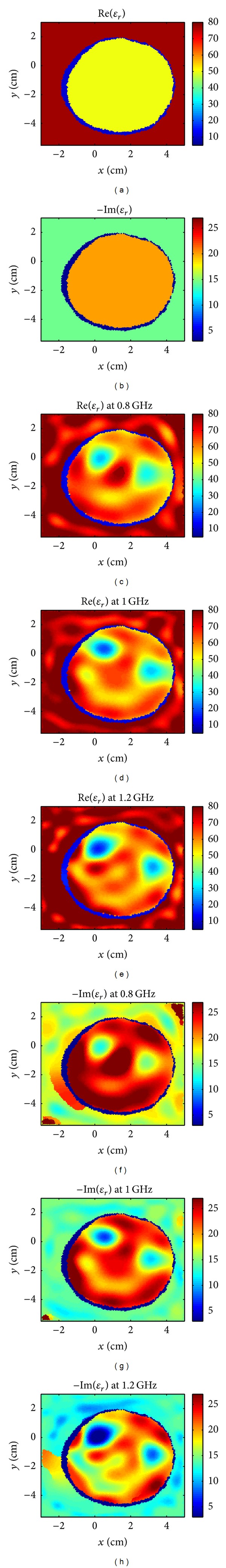
Volunteer 1: (a)-(b) prior information. The reconstruction when prior information is used as inhomogeneous background at (c), (f) 0.8 GHz, (d), (g) 1 GHz, and (e), (h) 1.2 GHz.

**Figure 11:**
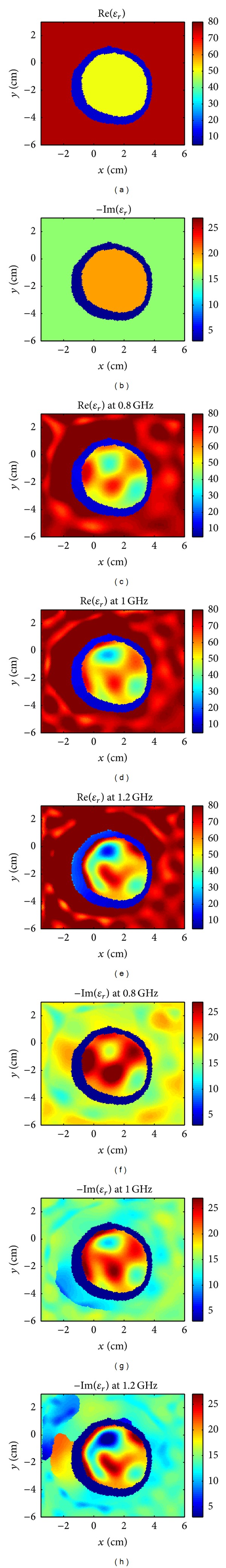
Volunteer 2: (a)-(b) prior information. The reconstruction when prior information is used as inhomogeneous background at (c), (f) 0.8 GHz, (d), (g) 1 GHz, and (e), (h) 1.2 GHz.

**Figure 12:**
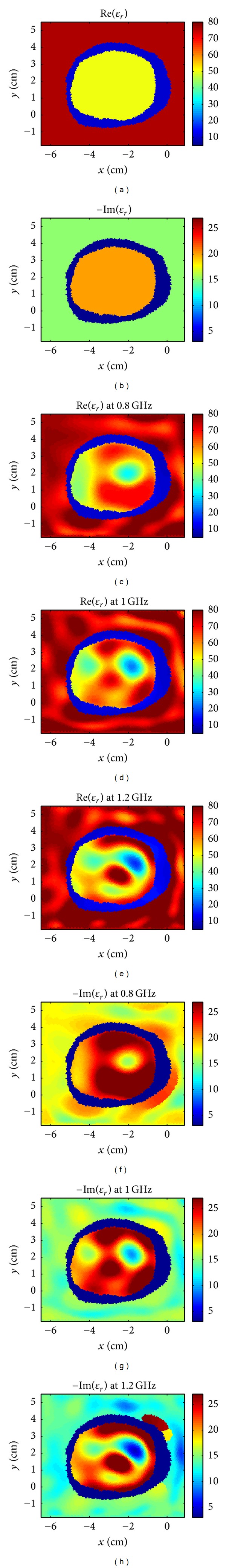
Volunteer 3: (a)-(b) prior information. The reconstruction when prior information is used as inhomogeneous background at (c), (f) 0.8 GHz, (d), (g) 1 GHz, and (e), (h) 1.2 GHz.

**Figure 13:**
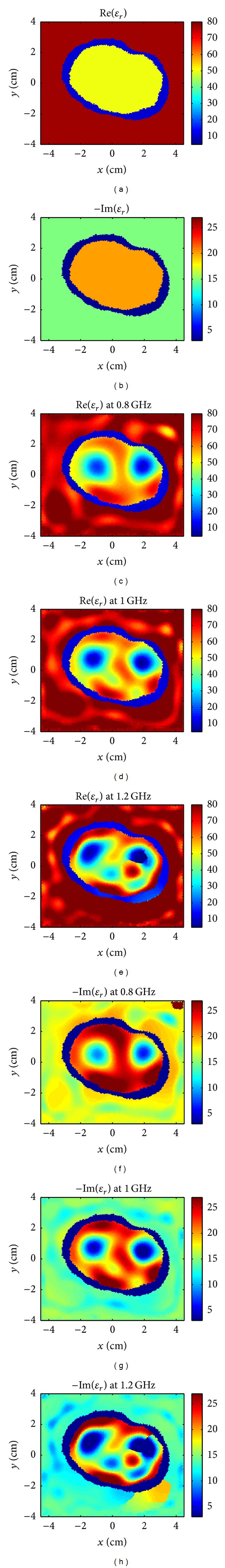
Volunteer 4: (a)-(b) prior information. The reconstruction when prior information is used as inhomogeneous background at (c), (f) 0.8 GHz, (d), (g) 1 GHz, and (e), (h) 1.2 GHz.

**Figure 14:**
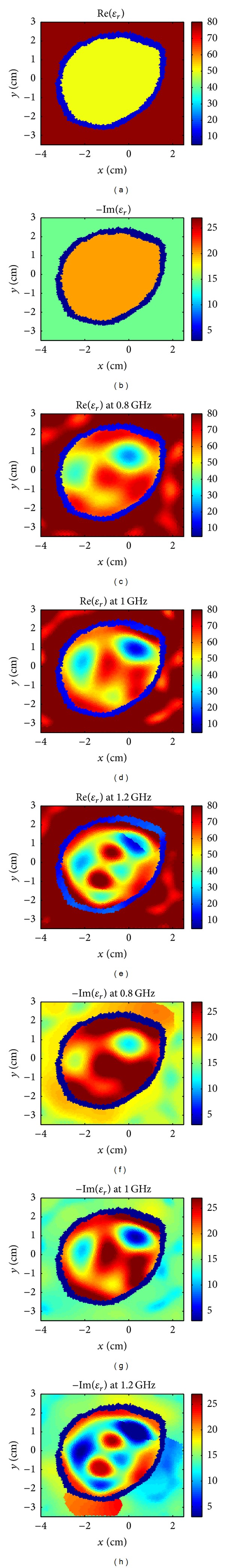
Volunteer 5: (a)-(b) prior information. The reconstruction when prior information is used as inhomogeneous background at (c), (f) 0.8 GHz, (d), (g) 1 GHz, and (e), (h) 1.2 GHz.

The inversion results at 0.8, 1, and 1.2 GHz are shown in Figures 10, 11, 12, 13, and 14. For each volunteer, the real and imaginary components of the estimated prior information are shown in subfigures (a) and (b) and the inversion results utilizing the prior information as inhomogeneous background in subfigures (c)–(h).

### 6.2. Discussion

For all volunteers, the use of the adipose layer as inhomogeneous background resulted in a substantially improved reconstruction of the forearms: the two bones can be clearly identified in all figures. The algorithm preserved the adipose layer used as prior information. With respect to the muscle tissues, the mean of the reconstructed dielectric values is close to values from the blind inversion in Section 5. As for the variations within the muscle regions, we speculate that they are due to the presence of other tissues (e.g., nerves, blood vessels, tendons, and connective tissues) in the forearm; these features can be observed in the MRI image. These improvements are of course only qualitative but clearly show the correct anatomical structure of the forearm. We have undertaken a controlled quantitative study, using synthetic numerical phantoms, of the improvements that are obtained with the use of prior information in the form of a known adipose layer. This study confirms that improvements in image quality are possible, but details are not included herein.

Artifacts, outside the forearms, are visible in the imaginary part reconstruction of several volunteers, for example, Figure 10(f) for volunteer 1 and Figure 11(g) for volunteer 2. These artifacts may be due to an error in estimating the adipose layer thickness and/or the location of the outer forearm boundary. In addition, the dielectric values used as prior data are *ex vivo* values taken from the literature, which may not be the same as the *in vivo* permittivity values [38]. Furthermore, the reconstruction of the left bone for volunteer 3, Figures 12(c) and 12(d), is not as good as the right bone; again the reason could be an error in the estimation of the adipose layer thickness.

For all the volunteers, the reconstructions at 1.2 GHz are not good compared to the results at 0.8 and 1 GHz. We believe that the reason is due to the higher noise level in the measurements at 1.2 GHz in comparison to the other two frequencies. As shown in Table 2, the calculated noise metric, *N*, is largest at 1.2 GHz for all the volunteers.

## 7. Conclusion

To the best of our knowledge, we have presented in this paper the first study of microwave limb imaging with multiple individuals (either human or animal). The use of an MRI image of the same transverse plane of the forearm as the microwave image has allowed us to determine the importance of adipose tissue with respect to the quality of the microwave images. Without the use of prior information, the microwave image quality is good only when the thickness of the adipose tissue is low.

The MWI reconstructions are improved when incorporating prior information as inhomogeneous background in the inversion algorithm; the internal anatomical features of all the volunteers' forearms with varying adipose tissue thickness were resolved. While the estimation of the prior information was performed manually using an *ad hoc* method, it improved the reconstruction results significantly in comparison to the blind inversion.

Supplanting the *ad hoc* technique used to extract prior information from the *blind* inversions of the experimental datasets with automated techniques is a topic for our future work. Preliminary investigations have revealed that it is possible to perform this information extraction automatically using image processing techniques, that is, postprocessing the blind inversion images. Alternative methods may also be possible such as the use of simple calipers to obtain the thickness of the adipose tissue in conjunction with methods to locate the boundary of the arm within the imaging domain, for example, using radar- or laser-based techniques.

## Figures and Tables

**Table 1:** Volunteer data.

Number	Sex	Age	Forearm
	(M/F)	(years)	(left/right)
Volunteer 1	M	32	Right
Volunteer 2	F	30	Right
Volunteer 3	F	48	Left
Volunteer 4	M	47	Right
Volunteer 5	M	42	Left

**Table 2:** Noise metric *N* of experimental data.

	0.8 GHz	1 GHz	1.2 GHz
Volunteer 1	24.7%	21.1%	31.0%
Volunteer 2	15.4%	15.4%	23.7%
Volunteer 3	27.8%	22.6%	30.0%
Volunteer 4	17.6%	18.8%	26.0%
Volunteer 5	22.2%	21.9%	33.0%

**Table 3:** Measured results from MRI images and estimates of lowest/highest relative permittivities from MWI in the muscle region at 1 GHz.

	MRI			MWI	
	Max. adipose	Arm width	Ratio	Min/Max of	Min/Max of
	Thickness (mm)	(mm)		*Re*(*ϵ*_*r*_)	− Im(*ϵ*_*r*_)
Volunteer 1	3.9	64.6	16.6	56/68	22/24
Volunteer 2	8.7	53.8	6.2	31/68	22/28
Volunteer 3	7.1	60.9	8.5	33/70	18/28
Volunteer 4	7.0	63.0	9.0	34/60	20/26
Volunteer 5	4.3	53.1	12.3	29/66	23/28
